# Optimizing tobacco treatment delivery for people with HIV: trial protocol for a randomized controlled trial

**DOI:** 10.1186/s13722-022-00341-2

**Published:** 2022-11-05

**Authors:** Brandon T. Sanford, Benjamin A. Toll, Allison Ross Eckard, Katherine R. Sterba, K. Michael Cummings, Nathaniel L. Baker, Alana M. Rojewski

**Affiliations:** 1 Department of Public Health Sciences, Medical University of South Carolina, 135 Cannon Street, MSC 835, 29425 Charleston, SC USA; 2 Hollings Cancer Center, Charleston, SC USA

**Keywords:** Implementation science, Tobacco treatment, HIV, Special populations, Opt-out, Smoking cessation

## Abstract

**Background:**

With advances in antiretroviral therapy, people with HIV (PWH) are living longer and are less likely to die from AIDS-related complications. Yet, prior research has shown that smoking is often not addressed in the context of HIV care, and few individuals are offered cessation treatment. Optimizing tobacco treatment delivery for PWH may increase engagement with evidence-based treatments and successful quit attempts.

**Methods:**

The current study is a type 1 hybrid effectiveness-implementation trial to evaluate the impact of a proactive, opt-out tobacco treatment intervention on cessation outcomes and advance understanding of key barriers and facilitators of implementation processes. A total of 230 PWH who smoke will be recruited from an infectious diseases clinic at an academic medical center and will be randomized to receive (1) treatment as usual (TAU) or (2) Proactive Outreach with Medication Opt-out for Tobacco Treatment Engagement (PrOMOTE). Primary outcomes include: biochemically verified 7-day point prevalence abstinence (PPA) rates, continuous abstinence (Weeks 9–12), and the number of 24-hour quit attempts at the end of study treatment (Week 12). Secondary outcomes include: participant reach (proportion reached out of contact attempts), implementation fidelity (including number of prescriptions written), participant adherence to prescribed pharmacotherapy, acceptability (participant and provider satisfaction with intervention delivery and content), and perceived barriers.

**Discussion:**

This study will examine a novel approach to optimizing tobacco treatment delivery for PWH. Integrating effectiveness and implementation results will help define best practices for engaging PWH with evidence-based tobacco treatment interventions. The intervention is low-cost, has the potential to be highly scalable, and could be translatable to other ambulatory HIV clinic settings.

**Trial Registration:**

ClinicalTrials.gov: NCT05019495 (August 24, 2021).

## Background

With advances in antiretroviral therapy (ART), people with HIV (PWH) are living longer and are less likely to die from AIDS-related complications. PWH are more likely to smoke than people without HIV,[1] and PWH lose more years of life to smoking than to HIV-related morbidity.[2] Smoking rates among PWH are approximately 30–50%,[3–5] which is nearly triple the 14% rate in the general United States population[6], and a majority report moderate or heavy nicotine dependence[7, 8]. The profound health effects of smoking are resulting in premature death from lung cancer and other smoking-related illnesses; lung cancer is now one of the leading causes of cancer death among PWH.[9, 10] Indeed, these increased rates of morbidity and mortality have even been detected in ART-adherent individuals,[10] and lung cancer incidence remains elevated for PWH for more than 5 years after smoking cessation.[5] Thus, provision of tobacco treatment is both imperative and time sensitive for PWH.

Clinical practice guidelines for treating tobacco use indicate that evidence-based pharmacological and behavioral interventions are recommended for all individuals who use tobacco, including PWH.[11–14] Several tobacco treatment pilot trials have been conducted in PWH,[15–20] but only a few large randomized controlled trials have been published.[21–23] Most studies that have evaluated pharmacotherapies have evaluated single-product NRT in PWH, with variable quit rates.[24] Many PWH have significantly faster nicotine metabolism than those without HIV, which could result in lower responsiveness to NRT.[25]. This difference in nicotine metabolism may underlie previous studies with PWH showing poor quit rates with NRT, particularly with single product NRT. Higher doses of NRT, which can be accomplished through dual product NRT (e.g., patch + lozenges),[13] or alternative pharmacotherapy approaches (e.g., varenicline) may be required to address these unique needs in PWH. Few studies have evaluated varenicline abstinence rates in PWH, but the research that has been conducted has shown that varenicline may be effective for PWH during treatment. Long-term efficacy remains inconclusive. Two placebo-controlled, randomized trials have been conducted to date that have shown varenicline to be efficacious and safe in PWH.[26, 27] Optimizing varenicline reach and clinical follow-up to improve adherence may help curb lapses/relapses and improve efficacy.[28–30].

Given that safety and preliminary efficacy of first-line pharmacotherapy in PWH have been demonstrated,[20, 21, 23, 26, 31] randomized controlled trials that evaluate methods for incorporating these medications into routine HIV clinic care to optimize access and use are needed. An evaluation of HIV Medical Association providers’ beliefs and practices found that providers generally agreed that smoking is an important issue in PWH, but only 41.7% and 46.0% of providers agreed that they frequently prescribed varenicline and NRT, respectively.[32] Provider prescribing practices are only one type of systemic barrier that PWH may encounter with respect to accessing tobacco treatment.[33] More specifically, in the context of HIV medical care, providers report that their greatest barriers to providing smoking cessation interventions are lack of time and feeling insufficiently confident to address smoking.[34] As such, even if PWH are frequently in touch with a prescribing provider, the lack of smoking cessation counseling, pharmacotherapy delivery, and follow-up support may prevent them from making a successful long-term quit attempt.

One strategy to address these barriers includes proactive referrals to trained tobacco treatment specialists who can tailor counseling and medication therapies for patients. The effectiveness of proactive smoking cessation approaches has been demonstrated in the literature at the population level and in clinical subspecialities. [33, 35–39] One large population-level trial of 5,123 participants found that proactive outreach for tobacco treatment resulted in greater 6-month prolonged smoking abstinence rates at 1 year compared to usual/reactive care (13.5% vs. 10.9%, respectively; p = .02).[36] In addition to proactive outreach, treatment opt-out approaches[37] have been shown to be effective for increasing the likelihood of receiving tobacco treatment pharmacotherapy, as well as increasing the odds of downstream smoking cessation.[40] Many hospitals and clinics currently utilize an opt-in approach, which may limit the reach of and access to tobacco treatment for PWH. A proactive, opt-out approach to tobacco treatment for PWH holds promise as a method for optimizing reach and access to tobacco treatment and successful smoking cessation.

In summary, previous research has emphasized the need for increasing the reach and delivery of evidence-based tobacco treatment for PWH. Herein we describe a study to evaluate the impact of a proactive, opt-out smoking cessation intervention which combines behavioral counseling and pharmacotherapy on cessation outcomes and advance understanding of key barriers and facilitators of implementation processes. Integrating effectiveness and implementation results will help define best practices for engaging PWH with evidence-based tobacco treatment interventions.

## Methods and analysis

**Fig. 1:**
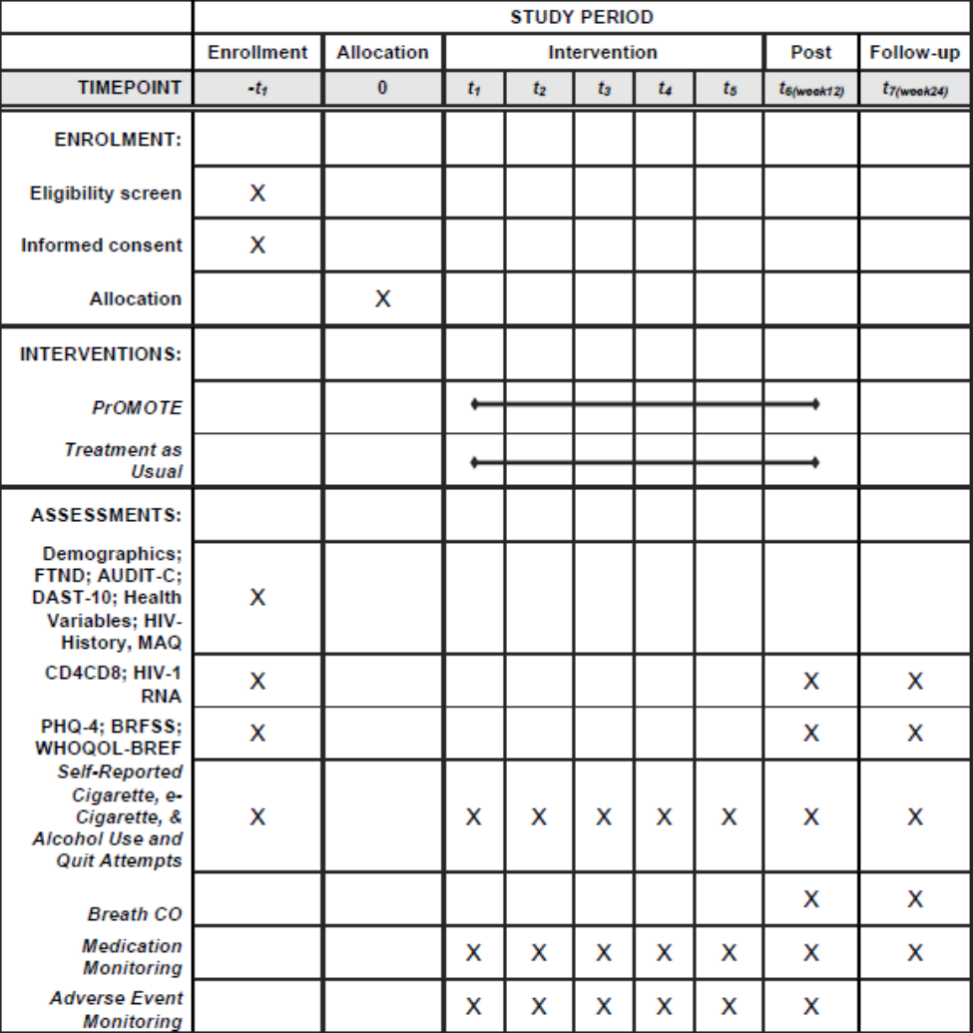
SPIRIT Flow Diagram

This clinical trial protocol follows the Standard Protocol Items: Recommendations for Interventional Trials guidelines,[41] and is registered on ClinicalTrials.gov (NCT05019495; ClinicalTrials.gov: NCT05019495 (August 24, 2021). Study enrollment began on December 1, 2021, and the estimated primary completion date is September 30, 2025. The schedule of enrollment, interventions, and assessments is presented in the SPIRIT flow diagram (Figure 1) .

### Participants

The target sample will be 230 PWH who currently smoke. Inclusion criteria include: (1) age 21 and older, (2) current diagnosis of HIV-1, (3) current smoking (defined as self-report of current smoking), (4) English speaking; and (5) willingness to be randomized into treatment and/or control conditions. Exclusion: 1) currently imprisoned.

### Recruitment

Potential participants will be recruited and screened for inclusion and exclusion criteria from an infectious diseases clinic at an academic medical center. Research staff will review the clinic schedule 3 weeks in advance and will send a letter to potentially eligible patients prior to their appointment. The letter will inform them that they may be eligible to participate in a research study and that research staff will be reaching out to them prior to their next clinic appointment. They will be instructed to call the research team to opt-out of the informational call. If they do not opt-out, the research staff will call these patients prior to their appointment to explain the study and enroll the patients if they are interested in participating. The research staff will meet the patient via an approved telehealth video platform.

### Consent

Informed consent and HIPAA authorization will be obtained prior to any other procedures. Informed consent will be obtained either via REDCap electronic consent (e-consent) combined with a phone or video discussion, by mail combined with a phone discussion, or in person.

### Randomization

Participants will be randomized to a treatment assignment (treatment as usual [TAU] or Proactive Outreach with Medication Opt-out for Tobacco Treatment Engagement [PrOMOTE]) in a 1:1 allocation. Randomization will be done using a stratified random block design and will be stratified across biological sex at birth and motivation to quit (motivation ladder; low: 0–7 vs. high: 8–10). Participants randomized to PrOMOTE will be scheduled to see the Tobacco Treatment Program clinical pharmacist. Those randomized to TAU will follow traditional clinic pathways for receiving tobacco treatment in the infectious diseases (ID) outpatient clinic.

### Procedures

Participants who consent to participation and are randomized will complete baseline assessments and a blood draw for baseline measurements of CD4/CD8 cell counts and HIV-1 RNA (viral load). Participants will be compensated for completion of the baseline interview; transportation and testing will be paid by the study.

Treatment as usual (TAU)TAU participants will follow traditional clinic pathways for receiving tobacco treatment in the HIV outpatient clinic. This typically consists of patients seeing the ID clinical pharmacist (PharmD) for medication management (ART and any other medications to treat comorbidities). The clinical pharmacist can prescribe pharmacotherapy if a patient is a current smoker and is interested in quitting. Thus, the TAU approach is conducted in an opt-in fashion. However, all participants randomized to TAU will have the opportunity to access smoking cessation pharmacotherapy from the clinical pharmacist if they choose to opt-in.

Proactive outreach with medication opt-out for Tobacco Treatment Engagement (PrOMOTE): The treatment will consist of a proactive, opt-out, pharmacist-led smoking cessation counseling and pharmacotherapy intervention. All participants who are randomized to receive the PrOMOTE intervention will be proactively scheduled for a virtual (HIPAA-compliant video conferencing or telephone) session with the clinical pharmacist (PharmD). The clinical pharmacist will assess their smoking status, conduct a motivational interview (described below), and arrange a prescription for pharmacotherapy (described below). They will schedule the participants for two additional sessions (approximately every 4 weeks) to assess medication adherence, potential side effects, and provide additional cessation counseling or relapse prevention counseling as needed. The clinical pharmacist will also create a note in the participant’s electronic medical record documenting the clinical encounter, counseling content checklist, the medications that were prescribed and mailed, adverse events review, and updates about the participant’s smoking status.

*Brief counseling*: The clinical pharmacist will conduct a brief motivational interview and counseling session[13] that focuses on the benefits of quitting smoking, committing to a quit attempt, cigarette tracking to identify smoking cues, and behavioral strategies to manage cravings and stress. The counseling provided by the clinical pharmacist will be based on practical counseling, which is a cognitive-behavioral, evidence-based, smoking cessation treatment modality,[13] and motivational interviewing.[42] The counseling session will last approximately 30 min.

*Pharmacotherapy*: Based on the recent American Thoracic Society Clinical Practice Guidelines, preference will be given to prescribing varenicline over NRT.[12] Participants will be informed that they medically qualify for a 12-week prescription of varenicline. If they opt-out of varenicline use (or cannot take it due to medical comorbidities such as renal disease), the clinical pharmacist will offer dual NRT (nicotine patches and lozenges) or bupropion. If the participant opts out of any medication use, the pharmacist will conduct a brief motivational interview to encourage the use of smoking cessation pharmacotherapy. If the participant still elects to opt out, the clinical pharmacist will offer follow-up behavioral counseling. The medication opt-out language from the clinical pharmacist will be consistent for each participant and scripted: *“What I would like to do is prescribe you varenicline to help you quit smoking. I have reviewed your chart and varenicline is safe to use with your other medications and has been shown to reduce cravings to smoke. I reviewed your prescription coverage and this medication will be $__. If I can verify your address, I will mail this directly to you.”*

Participants will have their study drug covered by their health insurance and mailed to their home (or picked up at a pharmacy). This may include the AIDS Drug Assistance Program (ADAP) if they are uninsured, or 340B funds can be used to cover co-payments if the medication is not fully covered by their health insurance. All participants will be encouraged to start their medication as soon as they receive it and to set a quit date 1 week from medication initiation. The varenicline prescription will follow the standard titration schedule. Participants will remain on varenicline for 12 weeks total. Dose adjustments (e.g., reduction to 0.5 mg twice per day if 1 mg twice per day is not well-tolerated) will be allowed at the discretion of the clinical pharmacist. The dual NRT (or single NRT add-on to other pharmacotherapy such as varenicline or bupropion) prescription will follow standard dosing recommendations. Switching to the other pharmacotherapy (from varenicline to dual NRT or vice versa) will be permitted if the participant reports treatment failure or high frequency of side effects after 1 week of appropriate use, based on the clinical judgement of the pharmacist.

A novel alert system will be built in to the adverse event surveys (see “Assessments”) within the REDCap database such that any severe physical adverse events (e.g., severe nausea, severe sleep disturbances, severe changes in mood) that are endorsed along with self-report of smoking cessation pharmacotherapy use will trigger an alert to the pharmacist. This alert system is intended to improve varenicline (or NRT) medication adherence as the pharmacist can quickly follow-up on any side effects that may be associated with pharmacotherapy use and call the participant with any recommended dose adjustments.

Follow-up: Participants in both study arms will be asked to complete the following surveys: tobacco use calendar, use of pharmacotherapies, quit attempts, adverse events, and psychiatric events (see “Assessments” below) every 2 weeks after randomization. The assessments will be completed via participant interview by research staff over the phone or remotely via REDCap from a smartphone or a computer. Additional assessments will be completed at the Week 12 and Week 24 follow-ups. Participants will also complete a blood draw at Week 12 and 24 to measure CD4/CD8 cell counts and HIV-1 RNA viral load. Participants will be paid $50 for completion of each follow-up interview.

### Measures

Participants will complete a series of baseline measures at their first meeting by phone or through REDCap’s automated survey feature, including assessments of demographics, smoking behavior (including electronic cigarettes; behavioral risk factors (Behavioral Risk Factor Surveillance System [BRFSS][43]), alcohol (Alcohol Use Disorders Identification Test-C [AUDIT-C][44]) drug use and misuse (including marijuana[43]; Drug Abuse Screening Test-10 [DAST-10][45]), health, and HIV infection history. The Fagerström Test for Nicotine Dependence (FTND) will be completed at baseline to assess the severity of dependence on nicotine.[46–48] Participants will also verify their list of current medications, including ART regimen. Quantity and frequency estimates of tobacco, e-cigarettes, and alcohol use will be assessed using a Timeline Follow-Back Procedure starting 30 days prior to intake (repeated biweekly post-randomization until Week 12 and at the Week 24 follow-up).[49] Number of quit attempts and number of 24-hour quit attempts will be assessed using a similar calendar method biweekly from Weeks 2–12 and at the Week 24 follow-up. The Medication Adherence Questionnaire (MAQ) will be completed at baseline to assess each participant’s history of medication adherence.[50, 51].

 Participants will be assessed regarding their level of knowledge and attitudes toward varenicline and NRT at baseline and at Week 24. Additionally, every 2 weeks they will be asked if they received (PrOMOTE) or received/filled (TAU) their pharmacotherapy prescription (yes/no). The amount and frequency of smoking cessation pharmacotherapy used (per product) will be determined by utilizing the calendar method of data collection described above for tobacco use. Adherence to the smoking cessation pharmacotherapy regimen will also be calculated and will be defined as 80% or more of prescribed doses taken and will be calculated based on the self-report data from this assessment. Motivation and confidence to quit smoking will be assessed using a modification of the Contemplation Ladder[52, 53] which assesses readiness to quit in the next month.

Adverse events will be assessed every 2 weeks via REDCap, and participants will be asked to rate their self-reported physical health problems as “mild,” “moderate,” or “severe.” If participants endorse any of their symptoms as being “severe” in nature, and they also endorse using smoking cessation pharmacotherapy, REDCap will trigger a notification that is sent to the research staff and the clinical pharmacist who will review the report and assess whether follow-up with the participant is required. The alert can also be triggered if the individual requests a provider phone call to discuss mild to moderate symptoms that occur in conjunction with pharmacotherapy use.

Participants will be assessed for anxiety and depressive symptoms (Patient Health Questionnaire-4 [PHQ-4][54]), cognitive decline (BRFSS[43]), and quality of life (WHO Quality of Life-Brief Version [WHOQOL-BREF][55]) at baseline and 12-week and 24-week follow-up. Suicidal ideation that may arise due to changes in mood when quitting smoking will be monitored using a single item question: “In the past two weeks, have you been thinking about killing yourself?” If “yes,” REDCap will trigger a notification that is sent to a clinical psychologist on the Tobacco Treatment Program team.

A visual analog scale (VAS) will assess the proportion of ART doses taken in the past 30 days.[56] The instructions read: “Place a mark on the line below at the point showing your best guess about how much of your ART medication you have taken in the last month (e.g., 0% means you have taken no medication 50% means you have taken half your medication; 100% means you have taken every single dose of medication). The VAS will range from 0 to 100 in 10% intervals.

Biochemical verification of abstinence will be obtained at the Weeks 12 and 24 follow-up using breath carbon monoxide (CO) measurements.[57] A breath CO monitor will be mailed to the participants with instructions for use with a webcam if needed. At a prearranged time, participants will be sent a telehealth video link. Participants will virtually meet with research staff, and will complete the breath CO sample live so that it can be confirmed the participant provided the sample and reduce the chance of sample falsification. The CO device will also email a report of the CO test result to the research staff. If the participant does not have internet capabilities, and therefore cannot use these devices virtually, the breath CO sample will be collected in person.

### Primary outcomes

Primary outcomes of smoking abstinence are defined as biologically-confirmed, end of treatment (EOT) 7-day point prevalence abstinence (Week 12) as well as 4 week continuous abstinence (CA; weeks 9–12).[57] Daily smoking diaries coupled with a breath CO ≤ 5 parts per million (ppm) will be utilized to determine abstinence.[57] To assess number of quit attempts, participants will be asked how many quit attempts and how many 24-hour quit attempts they made concurrent with each tobacco use calendar entry. The number of 24-hour quit attempts during study treatment will be tabulated and assessed at EOT.

### Secondary outcomes

Secondary outcomes related to implementation and intervention experiences will be assessed using surveys, an implementation tracking checklist and interviews with participants and providers.

### Exploratory outcomes

Exploratory outcomes include 7-day PPA at study follow up (Week 24) as well as the effect of abstinence on CD4/CD8 cell counts and HIV-1 RNA viral load. Participants will be asked “Have you smoked a cigarette or used any type of combustible tobacco products in the past 7 days?” coupled with a breath CO ≤ 5 ppm to determine abstinence. CD4/CD8 cell counts and HIV-1 RNA viral load will be measured at study baseline and follow up.

### Implementation outcomes

Implementation outcomes will be assessed consistent with Proctor’s framework[58, 59] and with the RE-AIM framework.[60] This includes reach, fidelity and acceptability, as well as perceived barriers to these outcomes. Reach includes the number and type of participants randomized to PrOMOTE and TAU, the number of contacts made, answered, and completed. Chart review will be utilized to assess the calls completed in both arms. Fidelity to the PrOMOTE intervention will be assessed using an implementation tracking checklist. Research staff and the clinical pharmacist will complete this tool for each participant visit in PrOMOTE to assess the clinical encounter and length, counseling content checklist, the medications that were prescribed and mailed, adverse reactions review, and updates about the participant’s smoking status. The staff will utilize chart review to assess the prescriptions written for TAU participants, and this data will be compared to the PrOMOTE data. Participant and provider acceptability will be assessed using the 4-item Acceptability of Intervention Measure (AIM) administered at the end of the study.[61] Items are scored on a 5-point Likert scale, and the resulting scale score is the mean of responses. The intervention will be considered acceptable if the average score within each arm across respondents is greater than or equal to 4 (scale range = 1–5). Barriers to implementation will be assessed using the implementation tracking checklist to monitor provider perceptions of barriers faced in completion of intervention steps.

Participant and provider interviews: Qualitative data will supplement survey and tracking data. Semi-structured interviews will be conducted with aproximately 20–25 participants from PrOMOTE to gain in-depth understanding of intervention experiences. Specifically, participants who enrolled but opted out of prescription pharmacotherapy (n = 10), and participants who enrolled and did not opt-out of prescription pharmacotherapy (n = 10) will be recruited. Interviews will continue until no new meaningful insights are revealed and theme saturation is achieved.[62, 63] Using a semi-structured interview guide, participants will be interviewed about their perceptions regarding the acceptability of the approach of the intervention (proactive, opt-out pharmacotherapy, counseling frequency, telehealth contact), barriers to engaging in tobacco treatment, and impact on motivation to quit and cessation outcomes. The clinical pharmacists from the Tobacco Treatment Program (PrOMOTE) and the ID clinic (TAU) will also be interviewed. Using a parallel semi-structured interview guide, providers will be interviewed about intervention acceptability including their perceptions regarding workflow, fidelity, fit within the clinic environment, resources to support and sustain the intervention in practice, and perceived clinic implementation and participant barriers. All participants (PWH and providers) will complete informed consent before completing the interviews and will receive compensation. Interviews will last 30–45 min and will be audio-taped and transcribed for analysis.

### Power and sample size

This study is powered to estimate the efficacy of PrOMOTE compared to TAU on abstinence from smoking at EOT. Investigations on the efficacy of varenicline note increases in 7-day PPA in active varenicline treatment arms as compared to placebo (Week 12: 50.3% vs. 21.2%; Δ = 29.1% and 50.3% vs. 20.8%; Δ = 29.5%).[64, 65] Similarly, Ashare and colleagues completed a trial of varenicline compared to placebo in PWH and noted that varenicline was superior to placebo at the close of treatment (PPA: 28.1% vs. 12.1%; Δ = 16.0%).[26] Further, dual NRT (long term patch + *ad lib* NRT) was similarly efficacious for abstinence at 6 months post quit as varenicline [dual NRT: abstinent = 36.5% (95% Confidence Interval [CI] = 28–45) and varenicline abstinent = 33.2% [29–38]].[13] Sample size will be based on a conservative estimate of the effect sizes provided above (Δ = 17%). Thus, a TAU abstinence rate of 15% is anticipated with a PrOMOTE abstinence rate of 32% at EOT. To detect this clinically-relevant effect size with 80% power and a type 1 error rate of 5%, n = 95 participants will be randomized to each of the two treatment assignments. Continuous abstinence from study Week 9 through 12 (4 weeks) will also be assessed as a primary efficacy outcome. Estimates on the efficacy of varenicline as compared to placebo note significant increases in 4-week end of study abstinence in the active varenicline treatment arms as compared to placebo at the close of study treatment (Week 12: 44.0% vs. 17.7%[64]; Δ = 26.3% and 43.9% vs. 17.6%; Δ = 26.3%[65]). Based on preliminary data, TAU continuous abstinence rate of 10% is anticipated with a PrOMOTE abstinence rate of 25% at the Week 12 (Δ = 15%). To detect this effect size with 80% power and a type 1 error rate of 5%, n = 97 participants will be randomized to each of the two treatment assignments. The study design will employ remote intervention delivery and follow-up assessments reducing participant burden and enhancing data collection. This, combined with the flexibility of medication management using PrOMOTE, a moderate to low attrition rate at EOT (15%) is anticipated. Thus, n = 115 participants randomized to each study treatment assignment (n = 230 total) will provide adequate power at a 5% type 1 error rate to detect the anticipated effect sizes.

### Data safety monitoring

Data will be collected on a secure, password-protected, electronic Web-based form and sent to a secure database in REDCap. Only research staff have access to the database. Participants will be assigned a study ID to protect confidentiality. The principal investigator (PI) will be responsible for monitoring the data, assuring protocol compliance, and conducting the safety reviews. A Data Safety Monitoring Board (DSMB) has been convened and consists of 3 investigators and 1 statistician who are independent of the proposed trial and experienced in various aspects of the conduct of clinical trials for tobacco treatment and/or clinical care for PWH. The PI will be responsible for monitoring the data, assuring protocol compliance, and compiling the data for safety reviews. Every 6 months the DSMB will review: (1) recruitment, retention, and follow-up rates for the study and compare them to target rates, (2) occurrence of all adverse events delineated by severity grading and causal relationship to study, (3) rates of recruitment of women and minorities with respect to targets, and (4) any other data that will help in the assessment of the clinical trial. These reports will be generated by the data manager every 6 months, and reviewed by the PI prior to their submission to the DSMB. During the review process, the DSMB, PI, and study staff will meet, minutes will be kept, the report will be reviewed, and the DSMB will vote on whether the study should: (1) continue with recruitment unchanged; (2) continue with a protocol amendment; (3) stop the study pending further investigation. If protocol modification or study suspension is needed, the PI will immediately inform the institutional review board (IRB). DSMB comments will be documented and forwarded to the IRB at the time of the annual review and reapproval; study-related serious adverse events and unanticipated problems will be reported in real time to the local IRB, the National Cancer Institute, and other regulatory entities as required.

### Planned analyses

Baseline clinical and demographic characteristics will be collected and preliminary analyses will examine significant correlates of study outcomes. Characteristics found to be significantly associated with primary outcome measures will be included as covariates in the initial stages of adjusted model development. Exploratory analysis of the modifying effects of participant sex on study outcomes will be assessed through main effects and the interaction between sex and study treatment assignment. Where significant interactions are found, sex-stratified analyses will be conducted to determine what role sex plays in the effect of varenicline on smoking outcomes in PWH. All statistical analyses will be conducted using the intent-to-treat (ITT) principal and will be completed using SAS v9.4 software.[66].

Clinical efficacy hypothesis: The primary study outcome of interest are the efficacy of PrOMOTE as compared to TAU in achieving biologically confirmed 7-day PPA and 4-week CA from cigarettes at EOT in PWH. To test these hypothesis, logistic regression models utilizing the sandwich variance estimate[67] will be developed. An ITT analysis will be utilized assuming participants that were lost to follow-up and those who withdrew from the study are smoking. Additionally, a completer analysis (participants who complete the Week 12 assessment) will be compared to the ITT results.

Additionally, participants will report all treatment emergent 24-hour quit attempts during study treatment (through Week 12). A Poisson distribution will be assumed with a logarithm link function to assess the effects of PrOMOTE on the number of quit attempts made as compared to TAU. Over-dispersion due to a wider than expected distribution in discrete count models (from heterogeneity) can have a significant impact on parameter inference, thus when detected, a negative binomial (NB) distribution will be specified.

Further, changes in CD4 and CD8 cell counts and the proportion of participants with detectable vs. undetectable HIV-1 RNA viral load will be examined from study baseline to Week 12 and Week 24, and participants who do and do not achieve abstinence will be compared at each timepoint. Generalized linear mixed effects regression models with appropriate distributions will be utilized to make comparisons. Model covariates will include baseline characteristics that were associated with follow-up abstinence.

Secondary examination of implementation outcomes: Descriptive statistics will be used to characterize implementation outcomes at the participant and provider level on reach (number and type of participants randomized, the number of contacts made, answered, and completed), provider fidelity (number of contacts completed and number of prescriptions written), participant adherence (number of prescriptions filled), medication adherence (assessed as both a continuous variable of percentage of prescribed medication taken as well as binary yes/no ≥ 80% adherence achieved), and acceptability (average score on the AIM Survey). Implementation outcomes for PrOMOTE will be characterized, and contrasts will also be performed between randomized treatment study arms (PrOMOTE vs. TAU). Continuous and ordinal outcomes will be compared using a Wilcoxon Rank-Sum test statistic while categorical outcomes will be compared using a Pearson Chi-Square test statistic.

Qualitative analysis: Transcriptions of digital recordings of participant and provider interviews will be analyzed to identify key barriers and facilitators to implementation processes and outcomes using content analysis[68] with NVivo software[69] to identify, categorize, and contextualize theme patterns. Two independent coders will read and reread transcripts for all qualitative analyses, outlining and organizing key themes and sub-themes. Discrepancies will be discussed in team quality assurance meetings to develop consensus.

Data synthesis: After completing qualitative and quantitative data analyses independently, results will be synthesized to relate quantitative findings from surveys and tracking logs with qualitative data for the purpose of analysis and data triangulation.[70] Themes identified in qualitative data will be supplemented by patterns identified in quantitative findings to characterize provider and participant impressions.

## Discussion

This is the first study to test an opt-out, proactive tobacco treatment delivery approach with a focus on pharmacotherapy and behavioral support. The intervention is low-cost, has the potential to be highly scalable, and could be translatable to other ambulatory HIV clinic settings. This study will add to the literature on varenicline and NRT efficacy in PWH. Although varenicline and NRT have been independently shown to be effective for smoking cessation, there are very few studies evaluating these pharmacotherapies in PWH. Whether these approaches will improve quit rates for PWH in an ambulatory clinical setting is an empirical question. Of note, this trial can be conducted completely remotely using rigorous methods that have been well-established. An exploratory assessment of mediators and moderators of smoking cessation outcomes (i.e., nicotine dependence, medication adherence) is also an important feature of this study as these variables are understudied in PWH. We will examine implementation experiences concurrent with the trial to advance future dissemination of PrOMOTE.

## Data Availability

Not applicable.

## Abbreviations

ART: Antiretroviral Therapy.
PWH: People with HIV.
NRT: Nicotine Replacement Therapy (NRT).
TAU: Treatment as Usual.
